# A 30-Year-Old Carrier of Gaucher Disease with Multiple Myeloma

**DOI:** 10.1155/2019/6469196

**Published:** 2019-02-13

**Authors:** Juskaran Chadha, Tanmay Sahai, Joshua Schwimmer, Dana Shani

**Affiliations:** Lenox Hill Hospital, Northwell Health, New York, NY, USA

## Abstract

We are reporting a case of a 30-year-old male with no past medical history who presented with new onset of renal failure, anemia, and splenomegaly and was diagnosed with multiple myeloma. Given the splenomegaly and the patient's Jewish heritage, blood tests were done and the patient was found to be a Gaucher disease carrier. The association of Gaucher disease and multiple myeloma has previously been reported; however, we want to describe the case of a young Gaucher disease carrier who developed multiple myeloma and provide a review of the literature.

## 1. Introduction

Multiple myeloma (MM) is a clonal disease of the plasma cell, resulting in renal failure, anemia, hypercalcemia, and bone lesions. MM accounts for 1% of all malignancies and up to 10% of hematologic malignancies. In 2015, the annual incidence of MM in the United States was 26,850, with 11,240 deaths [1]. The median age at diagnosis is 65-70 years with increased incidence among African-Americans. The etiology of MM is unknown; however, radiation, exposure to benzene and other organic solvents, chronic antigen stimulation, and certain genetic factors including single nucleotide polymorphism (SNP) variants and HLA types have been linked to increased risk. The international staging system (ISS) is helpful for prognostication and includes measurements of beta-2 microglobulin and albumin levels. In addition, patients with del17p, t(4;14), t(14;16), and t(14;20) have poor prognosis. After risk stratification, patients undergo induction therapy, sometimes followed by autologous bone marrow transplant when indicated, followed by maintenance therapy.

## 2. Material and Methods

A 30-year-old male with no significant history who was recently treated with levofloxacin for a community-acquired pneumonia presented to the hospital with abnormal renal function and anemia. The patient presented with a normocytic anemia, hemoglobin of 9.3, creatinine of 3.10, and uric acid of 11.1. On CT imaging, the patient had splenomegaly up to 18 cm and innumerable osteolytic lesions, predominantly in the pelvis. The patient was subsequently found to have an IgG lambda M-spike of 6.5 gr/dl. Bone marrow biopsy was obtained and was consistent with plasma cell myeloma. Upon immunophenotypic analysis, monoclonal IgG lambda (CD38 bright) population was present at 15% of the bone marrow. Further evaluation on FISH and cytogenetics revealed a translocation t(11;14) gene rearrangement and del17p. Patient subsequently underwent treatment at an outside institution. Given his renal dysfunction, he was started on induction therapy with CyBorD (cyclophosphamide, bortezomib, and dexamethasone). Patient achieved partial response with improvement in organ dysfunction and was subsequently transitioned to D-RVD (daratumumab, bortezomib, lenalidomide, and dexamethasone). Patient achieved a very good partial response and had an M-spike of 0.1 gr/dl prior to transplant. He successfully underwent an autologous stem cell transplant with melphalan-200 and was recently discharged with a hemoglobin of 12.2, a creatinine of 0.98, and plans to initiate maintenance therapy. For his bony lesions, he has been tolerating therapy with denosumab.

Given the patient's Jewish heritage and presence of splenomegaly, testing for Gaucher disease was performed and the patient was found to be a carrier of Gaucher disease. Peripheral blood specimen was sent off revealing one copy of the c.1226A>G (p.Asn409Ser) mutation in GBA.

## 3. Discussion

Gaucher disease is an autosomal recessive metabolic disorder secondary to genetic deficiency of the lysosomal enzyme glucocerebrosidase, which allows for the accumulation of its natural substrate glucosylceramide and its deacylated product glucosylsphingosine in the lysosomes of macrophages. These macrophages are known as “Gaucher cells” [2]. In addition, carrier testing via assay of enzyme activity is unreliable due to the overlap of enzyme activity between carriers and noncarriers. Thus, identifying two disease-containing alleles in GBA provides confirmation of the diagnosis [3]. The most common type is Gaucher disease type 1, accounts for almost 95% of Caucasian patients [4]. Hematologic manifestations of the disease include anemia, thrombocytopenia, splenomegaly, and bleeding diathesis. Bone involvement causes stunted growth in childhood. Pulmonary involvement includes interstitial lung disease. There is no primary central nervous system involvement [5]. Acute Gaucher disease designated as type 2 involves the CNS including the brainstem and can lead to premature death. Subacute Gaucher disease, designated as type 3, also involves the CNS; however, systems appear later in life including ocular motor apraxia, spasticity, ataxia, seizures, and dementia.

Studies have noted an increased frequency in Gaucher disease with gammopathy and malignancy. The association with immunologic abnormalities and polyclonal hypergammaglobulinemia may occur at diagnosis in 14-41% of adults [6–9]. In Gaucher disease, the risk of multiple myeloma is 5.9 to 51.1 times higher compared to normal population [10]. Furthermore, lysosomal function, which is affected in Gaucher disease, includes priming of tissues for angiogenesis and metastasis formation [11]. The first reports of this association were made by Goldfarb and colleagues in 1950, where they found polyclonal hypergammaglobulinemia and Gaucher disease in a group of patients under the age of 30 [12]. In 2005, Rosenbloom and coworkers investigated the incidence of cancer in patients with Gaucher disease from patients enrolled in the International Gaucher Registry (ICGG). Data was analyzed on over 2,000 patients, but only 14% of the Gaucher population were older than 60 years. Ten patients were reported to have multiple myeloma, consistent with a relative risk of 5.9%, but there was no increase in risk for other types of cancer [13]. Interestingly, 68% of patients with Gaucher disease are of Jewish descent, most commonly Ashkenazi Jews [14]. They hypothesized that this elevated risk may be due to gene-linkage and coinheritance of proto-oncogenes adjacent to the GBA1 locus at 1q21 [15]. At this point, it is unclear whether GBA1 is itself a cancer gene or if carriers of GBA1 mutation have an elevated risk of cancer [16]. However, it has been theorized that one wild-type copy is sufficient to carry out normal cellular functions [17]. According to a recent review article by Stirnemann et al., carriers of GBA1 mutation have been predisposed to developing Parkinson's disease; however, risk of neoplasia is still uncertain [18].

The pathologic link between Gaucher disease and lymphoproliferative disorders may be related to enhancement in cytokine release [13]. In a further review of the literature, there have been theories that macrophages in Gaucher disease trigger B-lymphocyte immunoglobulin secretion via IL1/IL-6 secretion [19]. In addition, IL-10 level has been found to be higher than normal and can increase the occurrence of auto-antibodies and gammopathies [10, 20, 21]. Additionally, chronic long-term antigenic stimulation also could promote genomic instability [22]. In 2010, a mouse model was created with conditionally deleted GBA1 gene and subsequent analysis revealed significant dysfunction in the macrophages but also thymic T cells, dendritic cells, and osteoblasts in studying cytokine measurements and immunophenotyping [23]. Another hypothesis involves bioactive lipids. Both ceramide and sphingosine-1-phosphate are derived from glucosylceramide and glucosylsphingosine. Ceramide works to induce apoptosis while sphingosine-1-phosphate promotes development, cell growth, angiogenesis, and oncogenesis. Ultimately, the different ratio of these bioactive lipids may correlate with increased incidence of dysregulation of humoral immunity and B-cell malignancy in Gaucher disease [24, 25]. Further studies have shown that b-glucosylceramide 22 and glucosylsphingosine, which are two sphingolipids that accumulate in Gaucher disease, can be detected by a unique subset of type 2 natural killer T cells [26]. Moreover, these studies show that injection of these sphingolipids in vivo led to a rise in lipid specific type 2 natural killer T cells and subsequently the increase in downstream activation of B-cells and production of antibodies [27].

## 4. Conclusion

In summary, the association between Gaucher disease and malignancy is well-documented in the literature. Multiple studies have shown pathologic links between Gaucher disease and increased risk of malignancy, specifically multiple myeloma. Interestingly, the association of malignancy with Gaucher disease carriers still has not clearly been defined. Fortunately, the patient presented was successfully treated for his myeloma and is in clinical remission.

Given the paucity of literature on Gaucher disease carriers, this case report and the review of literature should serve to increase awareness of the potential for malignancy, especially hematologic malignancy, in patients who are Gaucher disease carriers.
